# The Female Pelvis Is Associated with a Lateralized Ischium and a Reduced Ischiofemoral Space

**DOI:** 10.3390/jcm12041603

**Published:** 2023-02-17

**Authors:** Sufian S. Ahmad, Christian Konrads, Marcel Niemann, Ulrich Stöckle, Henning Windhagen, Gregor M. Giebel

**Affiliations:** 1 Department of Orthopaedic Surgery, Hannover Medical School, 30625 Hannover, Germany; 2 Department of Orthopaedic Surgery, University of Tübingen, 72076 Tübingen, Germany; 3 Center for Musculoskeletal Surgery, Charité—University Medical Center Berlin, 13353 Berlin, Germany

**Keywords:** hip impingement, extra-articular hip impingement, hip pain, hip preservation, proximal femoral osteotomy

## Abstract

Background: Pelvi-femoral conflicts are increasingly recognized for their explanatory role in the pathology of extra-articular hip impingement. Ischiofemoral impingement (IFI) is a type of impingement between the femur and the ischium that causes high femoral antetorsion and valgus femoral neck orientation. It is unknown whether obstetric adaptation of the female pelvis renders the female hip at a higher risk of sustaining IFI. The aim of this study was to determine the influence of the pelvic morphology on the ischiofemoral space (IFS). Methods: Plain radiographs of healthy individuals with no symptomatic hip disease were obtained in a functional standing position in a standardized manner and utilized for measurement of the interischial and ischiofemoral widths, subpubic angle, and centrum collum diaphyseal (CCD) angle. Linear regression was performed to determine the influence of morphometric measures on the ischiofemoral space. Results: Sixty-five radiographs (34 females and 31 males) were included. The cohort was stratified according to gender. Significant gender-related differences were noted regarding the ischiofemoral distance (31% increase in males, *p* < 0.001), pubic-arc angle (30% increased in females, *p* < 0.001), and the interischial space (7% increase in females, *p* < 0.001). CCD did not significantly differ between genders (*p* = 0.2). Factors influencing the IFS include the pubic-arc angle (β = −0.01 (CI −0.02–−0.00), *p* = 0.003), interischial distance (β = −0.11 (CI −0.23–−0.00), *p* = 0.049) and CCD (β = −0.06 (CI −0.09–−0.04), *p* < 0.001). Conclusions: Obstetric adaptation is associated with an increased subpubic angle that shifts the ischia laterally and away from the symphysis. The resultant reduction in the ischiofemoral space renders the female pelvis at a higher risk for a pelvi-femoral conflict, or more precisely, an ischiofemoral conflict, due to the reduced ischiofemoral space of the hip. The CCD angle of the femur was shown not to be gender specific. However, the CCD angle demonstrates an influence on the ischiofemoral space, rendering the proximal femur a target for corresponding osteotomies.

## 1. Introduction

Pathomorphologies of the hip and the pelvifemoral area have been described as being associated with painful hip disorders and osteoarthritis [1,2,3,4,5]. Some attention was given to the ischiofemoral space and its association with posterior impingement and painful compression of the soft tissue structures between the ischium and the femur [6,7,8,9,10,11,12,13]. The clinical interest was substantiated by the knowledge of the impact of femoral antetorsion on the ischiofemoral space, emphasizing the importance of appreciating femoral antetorsion in the overall workup of the hip. Hip-preserving surgeons have advocated derotational osteotomies to address this impingement entity.

Majority of studies on ischiofemoral impingement reported a high prevalence amongst female patients [14]. It was therefore the aim of this study to determine whether the morphology of the female pelvis influences the ischiofemoral space of the hip. The corresponding knowledge should allow for a better understanding of the pathomorphology of this bony conflict.

It was hypothesized that there is a direct correlation between the subpubic angle and the ischiofemoral space in a functional posture.

## 2. Materials and Methods

### 2.1. Data Collection

An institutional database was utilized for the acquisition of conventional radiographs that were used for limb alignment analysis. The included radiographs were created using long-leg standing X-rays, as described below. Gender and age were noted. Radiographs of patients who had undergone bony surgery around the pelvis and femur (e.g., osteotomies or arthroplasty) were excluded. Radiographs that did not allow for visualization of the pubic-arc region or the ischii due to the presence of gonadal shielding, were excluded. Two orthopedic surgeons independently performed all radiographic measurements. Both observers repeated the assessments after six weeks. For the second phase, the order of all radiographic images was randomized to eliminate any bias from the first reading.

### 2.2. Radiographs

Long-leg weight-bearing X-rays were obtained using a 1.3-m cassette (Global Imaging Baltimore, MD, USA) as described by Dror Paley. The patient had to stand in a bipedal stance in front of a long film cassette. The X-ray tube was positioned at a distance of 3.05 m. The magnification with this setup was 5%. A 25 mm steel ball was used for calibration. The X-ray beam was centered at the level of the knee joints.

It was ensured that the patellae were positioned in such a way that they were between both condyles, pointing forward.

**Figure 1:**
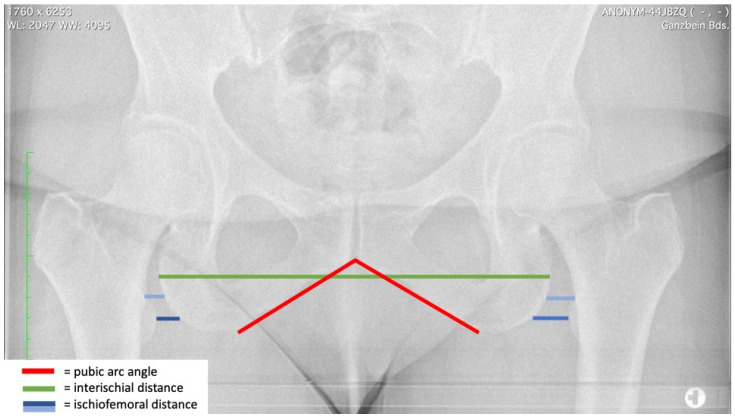
Illustration of the morphometric parameters. The red line represents the pubic-arc angle (also known as the subpubic angle). The green line represents the interischial space, defined as the distance between the lateral borders of both ischii. The blue lines (dark and light) represent the two lines used to measure the ischiofemoral distance.

Several radiographic measures were defined for the purpose of the study. The interischial distance is defined as the maximum horizontal distance between the lateral borders of both ischii on the AP view of the pelvis. Furthermore, the pubic-arc angle was measured. The ischiofemoral distance was measured as described in previous studies [15] (Figure 1). In brief, the distance measured between the femur and the ischium parallel to the horizontal pelvic orientation was measured based on two lines. The first line runs between the lateral cortex of the ischium and the most superior portion of the lesser trochanter; the second line runs parallel to the first between the ischium and the most medial point of the lesser trochanter (Figure 1). The centrum collum diaphyseal angle (CCD) and the lateral center edge angle (LCE) were also measured.

### 2.3. Statistical Analysis

Values were presented as the mean and standard deviation. A comparison was performed using Analysis of Variance (ANOVA), where the data was distributed parametrically. Linear regression was performed to test the influence of input variables on the ischiofemoral distance. A *p*-value of <0.05 was considered statistically significant. Statistical tests were performed using IBM SPSS Statistics version 27 (Armonk, New York, NY, USA).

We determined the interobserver agreement using the Pearson’s r interrater correlation coefficient for the radiographic measurements. For determining the intraobserver agreement, we calculated the mean kappa of both observers. These statistical analyses were performed using the software R, Vienna, Austria.

## 3. Results

The studied cohort consisted of 65 radiographs from 65 patients (34 females and 31 males). The mean age was 53 ± 10 years. The mean overall limb alignment was 3.3° ± 5° of varus on the right side. The mean CCD angle was 131.3° ± 7.3° on the right side. The mean pubic-arc angle was 101.5° ± 22.2°. The mean interischial distance was 16.4 cm ± 1.7 cm. The mean ischiofemoral space of the right hip was 2.3 cm ± 0.9 cm. The Pearson’s r interrater correlation coefficient for the radiographic measurements was 0.77, *p* < 0.001, for the interischial distance; 0.71, *p* < 0.001, for the ischiofemoral distance; and 0.81, *p* < 0.001, for the pubic-arc angle. The intrarater agreement was 95.1%.

Given an alpha of 0.05 and the above-mentioned sample sizes with the corresponding means and standard deviations of ischiofemoral space between both genders, post-hoc power was calculated to be 98.9%.

### 3.1. Gender Differences

**Figure 2:**
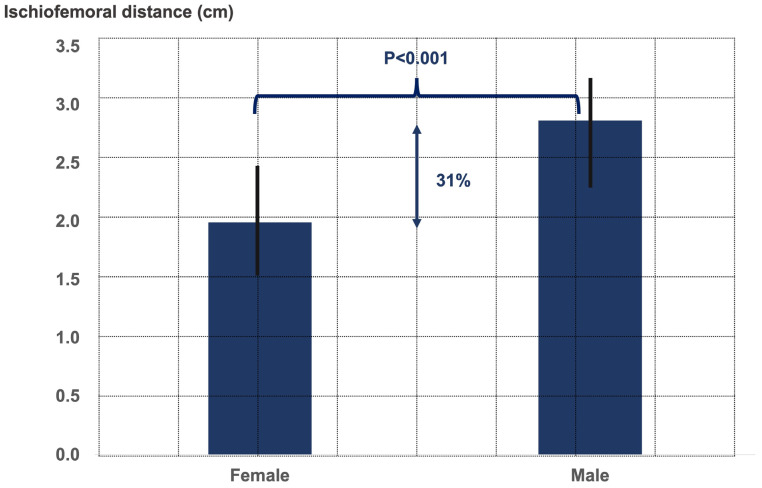
Graph illustrating the ischiofemoral distance in males and females.

**Figure 3:**
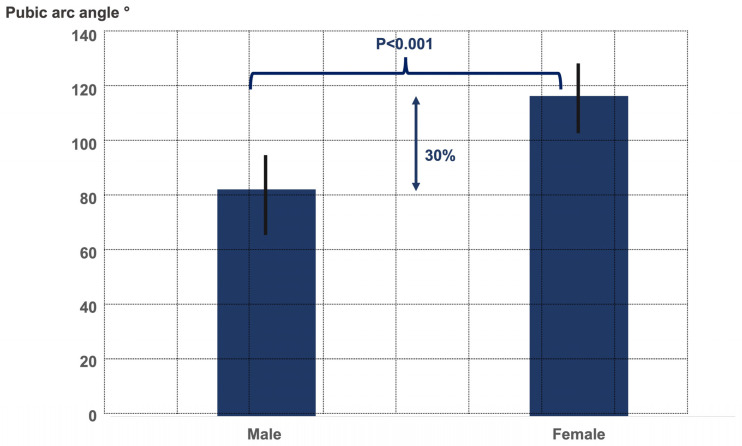
Graph illustrating the pubic-arc angle (subpubic angle) in males and females.

**Figure 4:**
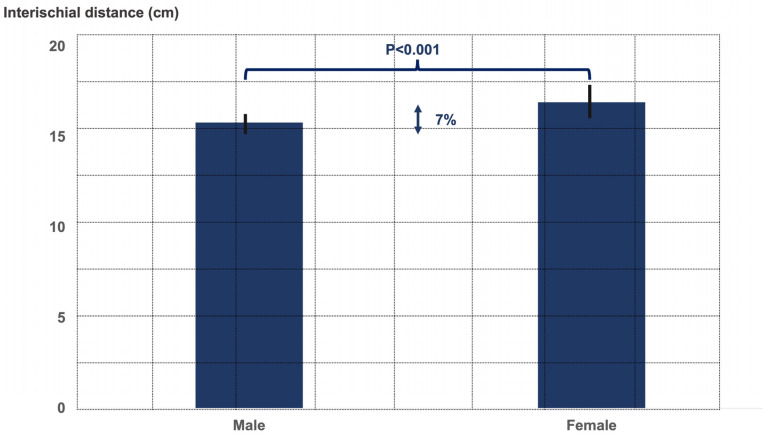
Graph illustrating the interischial distance in males and females.

The two gender groups did not significantly differ in terms of age (male age 54.0 ± 10.5 years, female age 52.8 ± 9.9 years, *p* = 0.6), limb alignment (male 2.5° ± 6.3° varus, female 4.0° ± 5.1° varus, *p* = 0.3), CCD angle (male 130.5° ± 7.2°, female 133.0° ± 7.3°, *p* = 0.2), and LCE angle (male 38.5° ± 6.5°, female 35.8° ± 6.6°, *p* = 0.4). There were significant differences between both gender groups regarding the ischiofemoral distance, the pubic-arc angle, and the interischial space (Figure 2, Figure 3 and Figure 4).

### 3.2. Regression Analysis

**Figure 5:**
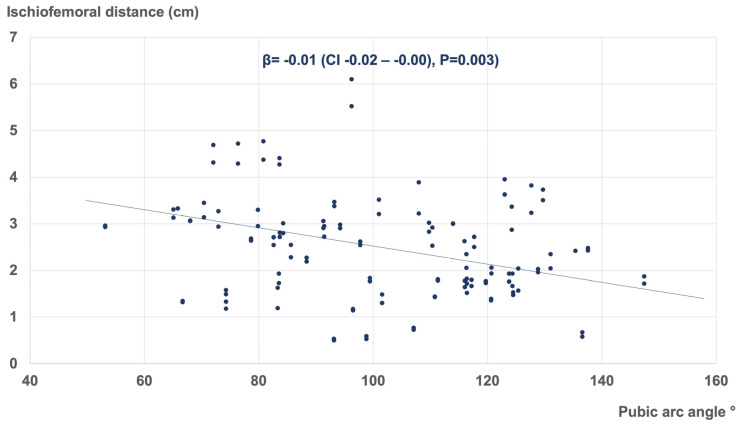
Linear regression demonstrating the association between the pubic-arc angle and the ischiofemoral distance (R^2^ = 0.82).

**Figure 6:**
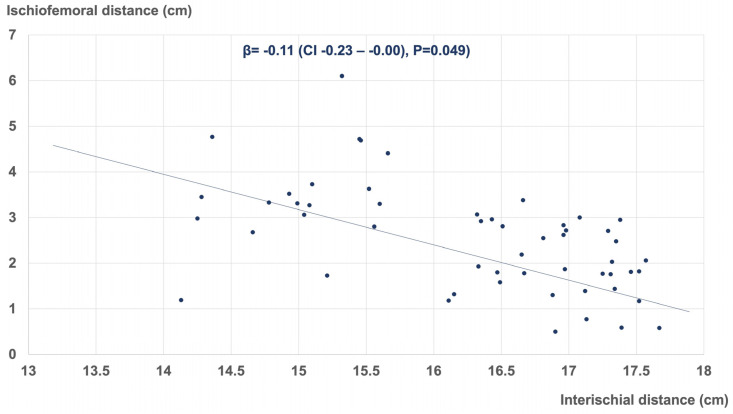
Linear regression demonstrating the association between the interischial distance and the ischiofemoral distance (R^2^ = 0.83).

**Figure 7:**
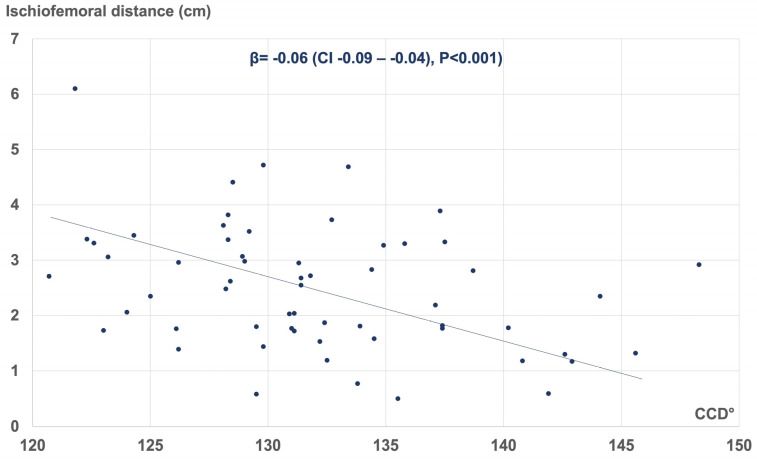
Linear regression demonstrating the association between the CCD angle and the ischiofemoral distance (R^2^ = 0.77). CCD: Centrum-Collum-Diaphyseal angle.

Multivariate linear regression analysis revealed several morphometric parameters influencing the ischiofemoral space of the hip. These included the pubic-arc angle (β = −0.01 (CI −0.02–−0.00), *p* = 0.003), interischial distance (β = −0.11 (CI −0.23–−0.00), *p* = 0.049), and CCD angle of the proximal femur (β = −0.06 (CI −0.09–−0.04), *p* < 0.001). Figure 5, Figure 6 and Figure 7 represent the corresponding scatter plots with the best fit lines.

## 4. Discussion

The most important finding of this study underlines the observation that there is a direct association between typical morphological features of the female pelvis and a reduced ischiofemoral space of the hip.

**Figure 8:**
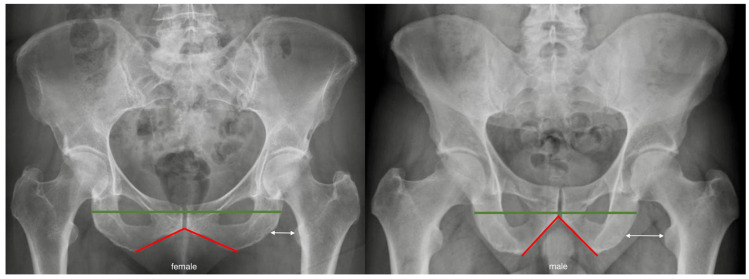
An example of a female pelvis on the left side and a male pelvis on the right. It is obvious that the increased pubic-arc angle (red) and corresponding widening of the interischial distance (green) results in a decrease in the ischiofemoral space (white), putting the female hip at a higher risk of ischiofemoral impingement.

The concepts of ischiofemoral conflicts and posterior hip impingement are gaining more recognition as a mechanistic explanation of hip pain. Interestingly, looking at the published reports of posterior hip impingement, it is obvious that the vast majority of reported cases were female. In a systematic review and meta-analysis by Singer, a total of 154 patients with ischiofemoral impingement were reported by five studies, of whom 133 (86.4%) were female and only 21 were male [14]. Furthermore, the handful of case reports demonstrating representative cases of ischiofemoral impingement have all reported female patients and no males [16,17,18,19,20,21]. The results of this study provide an explanation for this distribution that can clearly be attributed to the pubic-arc angle (also known as the subpubic angle) of the pelvis, which is inherently larger in females due to the obstetric adaptation of the female pelvis [22,23,24]. This ultimately increases the distance between both ischii and shifts the ischii closer to the femur (Figure 8). The ischiofemoral distance is therefore reduced, and the risk of impingement may therefore be increased. This study provides evidence to confirm this phenomenon.

It was demonstrated earlier that the ischiofemoral distance measured on ap radiograph correlates with the real three-dimensional ischiofemoral distance measured on transverse CT slides [2,15,25]. Yet, it is reasonable to mention that therapeutic decisions and surgical indications should be based on measurements on transverse imaging slides (CT or MRI).

There is limited therapeutic value in altering the pelvic morphology, which was shown to be a determinant of the ischiofemoral space. This brings the focus to the femoral side. One morphometric measure that directly influences the distance between the ischium and the femur is the CCD angle of the femur. A valgus orientation of the femoral neck is associated with a reduction of the distance between the ischium and the femur, while a varus orientation of the neck is associated with an increase in that space. Therefore, a proximal (varus-) osteotomy of the femur could be a viable therapeutic option for increasing ischiofemoral space and resolving a conflict. The clinical results of such osteotomies need evaluation in further studies.

This study showed a primary focus on the frontal plane. However, there are several studies highlighting the influence of femoral antetorsion on the orientation of the femoral neck in the axial plane and the corresponding influence on the ischiofemoral space [26]. There have been reports of proximal derotational osteotomies of the femur to reorient the position of the femoral neck in the horizontal plane [6]. The findings of this study emphasize the importance of considering proximal femoral variation in patients with severely reduced femoral offset.

There are several limitations to this study, one of which is associated with the two-dimensional nature of the study. Femoral torsion was not considered here but was well examined in prior studies; therefore, the results here are sufficient for the statements made regarding the frontal plane. The second limitation is the lack of clinical information that could have determined the occurrence of a symptomatic conflict. However, the aim of this study was more related to the morphology of the hip and pelvis than the symptomatic conflict itself.

This study is an initial work showing differences in pelvic anatomy by sex. Clinical decisions regarding treatment need to be made using three-dimensional imaging. Differences in pelvic anatomy were demonstrated based on two-dimensional imaging. Future studies correlating two-dimensional imaging with three-dimensional imaging are necessary to further validate the results presented here.

## 5. Conclusions

Obstetric adaptation is associated with an increased subpubic angle that shifts the ischia laterally and away from the symphysis. The resultant reduction in the ischiofemoral space renders the female pelvis at a higher risk for a pelvi-femoral conflict, or more precisely, an ischiofemoral conflict, due to the reduced ischiofemoral space of the hip. The CCD angle of the femur was shown not to be gender specific. However, the CCD angle demonstrates an influence on the ischiofemoral space, rendering the proximal femur a target for corresponding osteotomies.

## Data Availability

The data regarding this research article is part of the manuscript.

## References

[1] Ganz R., Parvizi J., Beck M., Leunig M., Nötzli H., Siebenrock K.A. (2003). Femoroacetabular impingement: A cause for osteoarthritis of the hip. Clin. Orthop. Relat. Res..

[2] Ahmad S.S., Kerber V., Konrads C., Ateschrang A., Hirschmann M.T., Stöckle U., Ahrend M.D. (2021). The ischiofemoral space of the hip is influenced by the frontal knee alignment. Knee Surg. Sports Traumatol. Arthrosc..

[3] Lerch T.D., Zwingelstein S., Schmaranzer F., Boschung A., Hanke M.S., Todorski I.A., Steppacher S.D., Gerber N., Zeng G., Siebenrock K.A. (2021). Posterior Extra-articular Ischiofemoral Impingement Can Be Caused by the Lesser and Greater Trochanter in Patients with Increased Femoral Version: Dynamic 3D CT–Based Hip Impingement Simulation of a Modified FABER Test. Orthop. J. Sports Med..

[4] Shon H.C., Park W.S., Chang J.-S., Byun S.-E., Son D.-W., Park H.J., Ha S.H., Park K.T., Park J.H. (2021). Long-term results of Bernese periacetabular osteotomy using a dual approach in hip dysplasia. Arch. Orthop. Trauma Surg..

[5] Hoch A., Schenk P., Jentzsch T., Rahm S., Zingg P.O. (2021). FAI morphology increases the risk for osteoarthritis in young people with a minimum follow-up of 25 years. Arch. Orthop. Trauma Surg..

[6] Lerch T.D., Schmaranzer F., Steppacher S.D., Ziebarth K., Tannast M., Siebenrock K.A. (2020). Most of patients with femoral derotation osteotomy for posterior extraarticular hip impingement and high femoral version would do surgery again. HIP Int..

[7] Torriani M., Souto S.C., Thomas B.J., Ouellette H., Bredella M.A. (2009). Ischiofemoral Impingement Syndrome: An Entity with Hip Pain and Abnormalities of the Quadratus Femoris Muscle. Am. J. Roentgenol..

[8] Carro L.P., Hernando M.F., Cerezal L., Navarro I.S., Fernandez A.A., Castillo A.O. (2016). Deep gluteal space problems: Piriformis syndrome, ischiofemoral impingement and sciatic nerve release. Muscle Ligaments Tendons J..

[9] Jo S., O’Donnell J.M. (2015). Endoscopic lesser trochanter resection for treatment of ischiofemoral impingement. J. Hip Preserv. Surg..

[10] Kivlan B.R., Martin R.L., Martin H.D. (2017). Ischiofemoral impingement: Defining the lesser trochanter–ischial space. Knee Surg. Sports Traumatol. Arthrosc..

[11] Lee S., Kim I., Lee S.M., Lee J. (2013). Ischiofemoral impingement syndrome. Ann. Rehabil. Med..

[12] Martin H.D., Khoury A., Schröder R., Palmer I.J. (2016). Ischiofemoral Impingement and Hamstring Syndrome as Causes of Posterior Hip Pain: Where Do We Go Next?. Clin. Sports Med..

[13] Stafford G.H., Villar R.N. (2011). Ischiofemoral impingement. J. Bone Joint Surg. Br..

[14] Singer A.D., Subhawong T.K., Jose J., Tresley J., Clifford P.D. (2015). Ischiofemoral impingement syndrome: A meta-analysis. Skelet. Radiol..

[15] Konrads C., Ahrend M.-D., Beyer M.R., Stöckle U., Ahmad S.S. (2022). Rotation osteotomy of the distal femur influences coronal femoral alignment and the ischiofemoral space. Arch. Orthop. Trauma Surg..

[16] Patti J.W., Ouellette H., Bredella M.A., Torriani M. (2008). Impingement of lesser trochanter on ischium as a potential cause for hip pain. Skelet. Radiol..

[17] Ali A.M., Whitwell D., Ostlere S.J. (2011). Case report: Imaging and surgical treatment of a snapping hip due to ischiofemoral impingement. Skelet. Radiol..

[18] Hackl M., Trost M., Boese C.K., Müller D., Eysel P., Dargel J. (2016). Bilateral Ischiofemoral Impingement: A Case Report. Z. Orthop. Unfall..

[19] Tosun Ö., Çay N., Bozkurt M., Arslan H. (2012). Ischiofemoral impingement in an 11-year-old girl. Diagn. Interv. Radiol..

[20] Hatem M., Martin H.D., Safran M.R. (2020). Snapping of the Sciatic Nerve and Sciatica Provoked by Impingement Between the Greater Trochanter and Ischium: A Case Report. JBJS Case Connect..

[21] Mimura T., Shibata K., Tamura S., Okumura N., Kumagai K., Maeda T., Mori K., Imai S. (2021). Ischiofemoral impingement between the ischium and the posterior facet of the greater trochanter: A case report. J. Orthop. Sci..

[22] Karakas H.M., Harma A., Alicioglu B. (2013). The subpubic angle in sex determination: Anthropometric measurements and analyses on Anatolian Caucasians using multidetector computed tomography datasets. J. Forensic Leg. Med..

[23] Huseynov A., Zollikofer C.P.E., Coudyzer W., Gascho D., Kellenberger C., Hinzpeter R., de León M.S.P. (2016). Developmental evidence for obstetric adaptation of the human female pelvis. Proc. Natl. Acad. Sci. USA.

[24] Young M., Ince J.G. (1940). A radiographic comparison of the male and female pelvis. J. Anat..

[25] Konrads C., Ahmad S.S., Histing T., Ibrahim M. (2022). Iatrogenic ischiofemoral impingement due to high tibial osteotomy with overvalgization: A case report. J. Med. Case Rep..

[26] Dablan A., Oktay C., Çevikol C. (2021). Ischiofemoral Impingement Syndrome: Effect of Morphological Variations on the Diagnosis. Curr. Med. Imaging.

